# Hospital Investment Decisions in Healthcare 4.0 Technologies: Scoping Review and Framework for Exploring Challenges, Trends, and Research Directions

**DOI:** 10.2196/27571

**Published:** 2021-08-26

**Authors:** Roberto Santiago Vassolo, Alejandro Francisco Mac Cawley, Guilherme Luz Tortorella, Flavio Sanson Fogliatto, Diego Tlapa, Gopalakrishnan Narayanamurthy

**Affiliations:** 1 IAE Business School Universidad Austral Pilar Argentina; 2 Department of Industrial and Systems Engineering Pontificia Universidad Católica de Chile Santiago Chile; 3 Department of Mechanical Engineering University of Melbourne Melbourne Australia; 4 Universidade Federal de Santa Catarina Florianopolis Brazil; 5 Departamento de Engenharia de Produção Universidade Federal do Rio Grande do Sul, Escola de Engenharia Porto Alegre Brazil; 6 Facultad de Ingeniería, Arquitectura y Diseño Universidad Autónoma de Baja California - Campus Ensenada Baja California Mexico; 7 Department of Operations and Supply Chain Management University of Liverpool Management School Liverpool United Kingdom

**Keywords:** healthcare 4.0, scoping review, investments, real options, health technology assessment, technological bundles, decision-makers, hospital, public health, technology, health technology, smart technology, hospital management, health care investment, decision making, new technologies

## Abstract

**Background:**

Alternative approaches to analyzing and evaluating health care investments in state-of-the-art technologies are being increasingly discussed in the literature, especially with the advent of Healthcare 4.0 (H4.0) technologies or eHealth. Such investments generally involve computer hardware and software that deal with the storage, retrieval, sharing, and use of health care information, data, and knowledge for communication and decision-making. Besides, the use of these technologies significantly increases when addressed in bundles. However, a structured and holistic approach to analyzing investments in H4.0 technologies is not available in the literature.

**Objective:**

This study aims to analyze previous research related to the evaluation of H4.0 technologies in hospitals and characterize the most common investment approaches used. We propose a framework that organizes the research associated with hospitals’ H4.0 technology investment decisions and suggest five main research directions on the topic.

**Methods:**

To achieve our goal, we followed the standard procedure for scoping reviews. We performed a search in the Crossref, PubMed, Scopus, and Web of Science databases with the keywords *investment*, *health*, *industry 4.0*, *investment*, *health technology assessment*, *healthcare 4.0*, and *smart* in the title, abstract, and keywords of research papers. We retrieved 5701 publications from all the databases. After removing papers published before 2011 as well as duplicates and performing further screening, we were left with 244 articles, from which 33 were selected after in-depth analysis to compose the final publication portfolio.

**Results:**

Our findings show the multidisciplinary nature of the research related to evaluating hospital investments in H4.0 technologies. We found that the most common investment approaches focused on cost analysis, single technology, and single decision-maker involvement, which dominate bundle analysis, H4.0 technology value considerations, and multiple decision-maker involvement.

**Conclusions:**

Some of our findings were unexpected, given the interrelated nature of H4.0 technologies and their multidimensional impact. Owing to the absence of a more holistic approach to H4.0 technology investment decisions, we identified five promising research directions for the topic: development of economic valuation methodologies tailored for H4.0 technologies; accounting for technology interrelations in the form of bundles; accounting for uncertainties in the process of evaluating such technologies; integration of administrative, medical, and patient perspectives into the evaluation process; and balancing and handling complexity in the decision-making process.

## Introduction

### Background

How do health care organizations manage and determine their investment decisions in Industry 4.0 (known as Healthcare 4.0 [H4.0]) technologies? Having the right answer to this question is essential because the health care value chain is increasingly applying H4.0 technologies [1]. In addition, the rising demand for more efficient, qualified, and less expensive health services has motivated novel technological solutions [2]. Health care organizations have incorporated innovative technologies around the internet to facilitate and support more efficient and flexible processes, services, and products [3,4]. Such technologies started playing a pivotal role as enhancers of efficiency and quality in health care systems in the 1990s, culminating in what is currently known as *eHealth* [5]. Health care institutions extend the emerging principles and technologies belonging to the Industry 4.0 realm to health care as a continuous and disruptive process of innovation and transformation of the entire health care value chain [6].

The magnitude of the technological shift, the scope of activities affected, and their interrelationships expose health care decision-makers to large and complex investment decision problems [7,8]. The scope of activities encompasses procedures, equipment, and processes used to deliver medical care [9]. The range of such investments usually involves computer hardware and software that deal with the storage, retrieval, sharing, and use of health care information, data, and knowledge for communication and decision-making [10]. Although it is possible to identify stand-alone technologies under the H4.0 umbrella, they tend to be highly interrelated, generating the need to assess them in bundles. In addition, there is significant uncertainty regarding which technology will be the industry standard, adding an extra level of complexity to financial evaluations.

As the level of investment required to stay competitive with these new technologies is massive, the financial budgets of health institutions and countries are constantly stressed. For instance, data from the BRICS nations (ie, Brazil, Russia, India, China, and South Africa) indicate that their average health expenditure grew from 5.41% of their gross domestic product in 1995 to 6.94% in 2013 and is forecast to reach an average of 7.86% by 2025 [11]. Hence, there is an increasing need for massive and interconnected investments that will impose nontrivial challenges in determining their value, optimum level, and implementation sequence.

Several different theoretical lenses help to enlighten managers in their technological investments. The Health Technology Assessment International Policy Forum recently concluded that the assessment paradigms need to be more agile, helping health care systems to understand the potential of innovations and ensure that their potential value is realized [12]. However, although the literature has suffered from balkanization because multiple alternative approaches have grown significantly in recent years, hospitals rarely have, or use, a systematic decision process for H4.0 technology investments, accounting for all organizational objectives and using objective data [13,14].

This paper aims to address the current gap between the literature and practice by examining trends, challenges, and research opportunities in hospital investment valuations of H4.0 technologies. To achieve this goal, we opted to carry out a scoping review of the literature, which is appropriate for identifying and mapping critical concepts that underpin a specific research topic, especially in the absence of previous comprehensive studies [15,16]. More importantly, the scoping review approach is also suggested as an alternative to a systematic review when the literature is vast, sparse, and complex [17,18], which is the case of investments in H4.0 technologies [19].

The paper has been structured as follows. First, we motivate the study, present the protocol for the scoping review (ie, the research method section), and summarize the manuscript selection process. Second, we define the research questions, identify the relevant studies, and select the final list. Third, we present the main findings in a section devoted to the analysis of results, addressing the first two research questions. Fourth, we develop a framework that synthesizes the analysis and identifies promising research directions regarding the most crucial characteristics for evaluating investments in H4.0 technologies, addressing the third research question.

### Hospital Investments, the Fourth Industrial Revolution, and Alternative Evaluation Approaches

The advent of Industry 4.0 technologies has significantly affected the global health care value chain. The recent integration of disruptive technologies derived from Industry 4.0 into health care systems aims at achieving virtualization to provide care in real time [20]. Health care institutions have incorporated cyberphysical systems, cloud computing, the Internet of Things, and big data, among others, into health care processes, services, equipment, material, and people. H4.0 technologies allowed the establishment of a smart system to monitor, track, and store patient records for ongoing care and analysis [21,22]. The combination of new technologies has expanded the scope of hospital activities. Economically, H4.0 technologies come with a value proposition of simultaneously improving efficiency and quality of care while reducing operating costs [23].

However, health care institutions need to carry out substantial investments to achieve the economic gains associated with H4.0 technologies. In 2014, US health care expenditure was US $3 trillion and is forecast to rise to US $5.1 trillion in 2023, outpacing the expected gross domestic product growth rate in the corresponding period [24,25]. These expenditures imply multiple investments that are not free of uncertainties because evaluating the impact on patient care is extremely difficult [26].

The unique characteristics of H4.0 technologies add a layer of evaluation complexity in an industry where assessing economic value is already challenging. For instance, studies on health technology assessment have primarily recognized that not every technological development results in net health gains [27]. The history of medicine and health includes many examples of technologies that did not produce the expected benefits or even proved harmful. At the same time, proving the effectiveness of technologies creates a continuous challenge for health systems because their application may require additional resources or compel health systems to choose from competing alternatives within the health system.

Studies have examined how health care organizations struggle to benefit from investments in H4.0 technologies [28,29]. Therefore, the dramatic increase in firms’ technology investments in recent years has not necessarily resulted in a significant increase in productivity [30]. The complexity involved in understanding the economic impact of H4.0 technologies resulted in nontrivial challenges in determining the policy and practice implications associated with them [31].

Organizations contribute significant financial resources to developing and implementing H4.0 technologies, and the potential for a negative return on investments or total implementation failure is a worrisome possibility [32]. Assessing technological investments is of great interest to hospital managers when they seek to raise capital to expand services [33]. With the rapid growth of eHealth in developing countries, there is an urgent need for substantial evidence of its impact on justifying and guiding the investment of resources in such systems [26].

Studies evaluating H4.0 technology investments have taken different approaches. A wide array of manuscripts focus on cost reduction evaluation. For instance, a study by Galani and Rutten [34] reported that health care decision-makers base their adoption decisions on cost-effectiveness and cost-minimization analyses. The main limitation of this approach is the focus on just one aspect of the decision (cost), underemphasizing value considerations.

The real-options approach to decision-making has been useful in capturing and valuing uncertainty in many operating decisions that decision-makers face [35]. Its utility lies in the fact that real options are contingent on future discretionary investment. The magnitude, timing, and schedule of the investment outlay affect the value of firms’ growth opportunities. Apart from correcting limitations from the cost perspective, the real-options approach increases the analytical effort that organizations need to carry out economic evaluations.

In addition, investing in H4.0 technologies requires multilateral stakeholder dialog and collaboration that address health needs and product conceptualization [12]. The nature of H4.0 technologies imposes challenges on how to assess the various aspects of technological value in the decision-making processes so that the assessment simultaneously accounts for the input of physicians, patients, and society [36].

Unsurprisingly, despite the expected benefits of H4.0 technologies and the interest from hospitals and policy makers in implementing them, the uptake and adoption of these technologies have not always been consistent within the health care practice, and adoption of these technologies has lagged [37]. There is a need to synthesize research activities and evidence to clarify the evaluation process of H4.0 technology investment in hospitals. Our scoping review explores this knowledge gap by mapping the extent and nature of the available literature and focusing on literature-based evidence that examined the integration of H4.0 technology investments into hospitals.

## Methods

### Overview

The scoping review design represents a methodology that allows the assessment of emerging evidence; therefore, it is the first step in research development [16]. It is a relatively new approach to evidence synthesis and differs from systematic reviews in its purpose and aims. The purpose of a scoping review is to provide an overview of the available research evidence without producing a summary answer to a discrete research question [38]. The methodology can help answer broad questions and gather and assess information before conducting a systematic review. It is suitable for achieving several objectives such as identifying the types of existing evidence in a given field, clarifying key concepts or definitions in the literature, surveying how research is conducted on a specific topic, identifying key characteristics related to a particular topic, and identifying knowledge gaps. Compared with systematic literature reviews and meta-analyses, a scoping review provides more flexibility and allows for diverse, relevant studies that use different methodologies [17,39,40]. Our research domain is adequate for performing a scoping review because studies regarding H4.0 technologies are multidisciplinary and relatively new.

To achieve our goal, we followed a standard scoping study procedure comprising five steps: (1) identify the research questions; (2) identify relevant studies; (3) select studies; (4) chart the data; and (5) collate, summarize, and report the results. In the following sections, we detail each stage and the outcomes of our study.

### Identify the Research Questions

As with most systematic literature reviews, scoping reviews start with a primary research question to focus the inquiry [15,16], guiding researchers to build the search strategies [17]. Our broad initial research question was “How have health care institutions assessed their H4.0 technology investments?” However, given the multidisciplinary nature of the subject and the comprehensive sources of the reports, we narrowed the main research question into three more specific research questions:

Research question 1: What methodologies do health care institutions use for evaluating investments in H4.0 technologies?

Research question 2: What are the main challenges faced by health care institutions when evaluating investments in H4.0 technologies?

Research question 3: Which are the most important characteristics that the methodologies for evaluating investments in H4.0 technologies must have?

To answer these questions, we developed a rigorously structured and sufficiently documented method to provide robust evidence and arguments.

### Identify Relevant Studies

A scoping review requires the identification of all relevant studies, regardless of the methodological design [16]. This step aims to find all available published and unpublished studies that address the research questions, operationalized through the search terms. As familiarity with the research topic is likely to increase as the review advances, we searched for relevant studies in two stages. In the first stage of identification, to include as many relevant studies as possible, we defined the set of keywords that best represented the scope of the study. In the second inclusion stage, we randomly selected a group of papers from each database and analyzed their keywords to determine the need to add more keywords to our inquiry. This two-stage process allowed us to address the search string’s potential problem of being overly specific or entailing (partially) misleading buzzwords.

In the first stage, we defined the three research dimensions or keywords that best reflected our research questions: investment, health, and Industry 4.0. Subsequently, we combined an initial set of keywords using the AND and OR Boolean search operators (*investment* AND *health*, *health* AND *industry 4.0*, *investment* AND *industry 4.0*, *health technology assessment* AND *industry 4.0*) to retrieve publications that used them in the title, abstract, and keywords. The use of the AND operator in the search process significantly reduced misleading results, especially in the case of the *4.0* string. We searched for scientific articles in the following databases: Crossref, PubMed, Scopus, and Web of Science (which comprises biomedical literature from MEDLINE, life science journals, and web-based books).

As H4.0 derives from principles and technologies from Industry 4.0, whose concept was formally acknowledged in 2011 [41], we only considered publications after that year. Furthermore, in the widely referenced literature review by Liao et al [3], the authors indicated that, although the announcement of the Industry 4.0 concept traces back to April 2011, it began to attract attention only after it became one of the ten official projects within the *High-Tech Strategy 2020* action plan in March 2012. In fact, no study was identified before that date, supporting the choice of the cut-off year of 2011 for our scoping review.

We applied the query string to the indicated databases and retrieved a total of 5701 publications from these databases.

**Figure 1:**
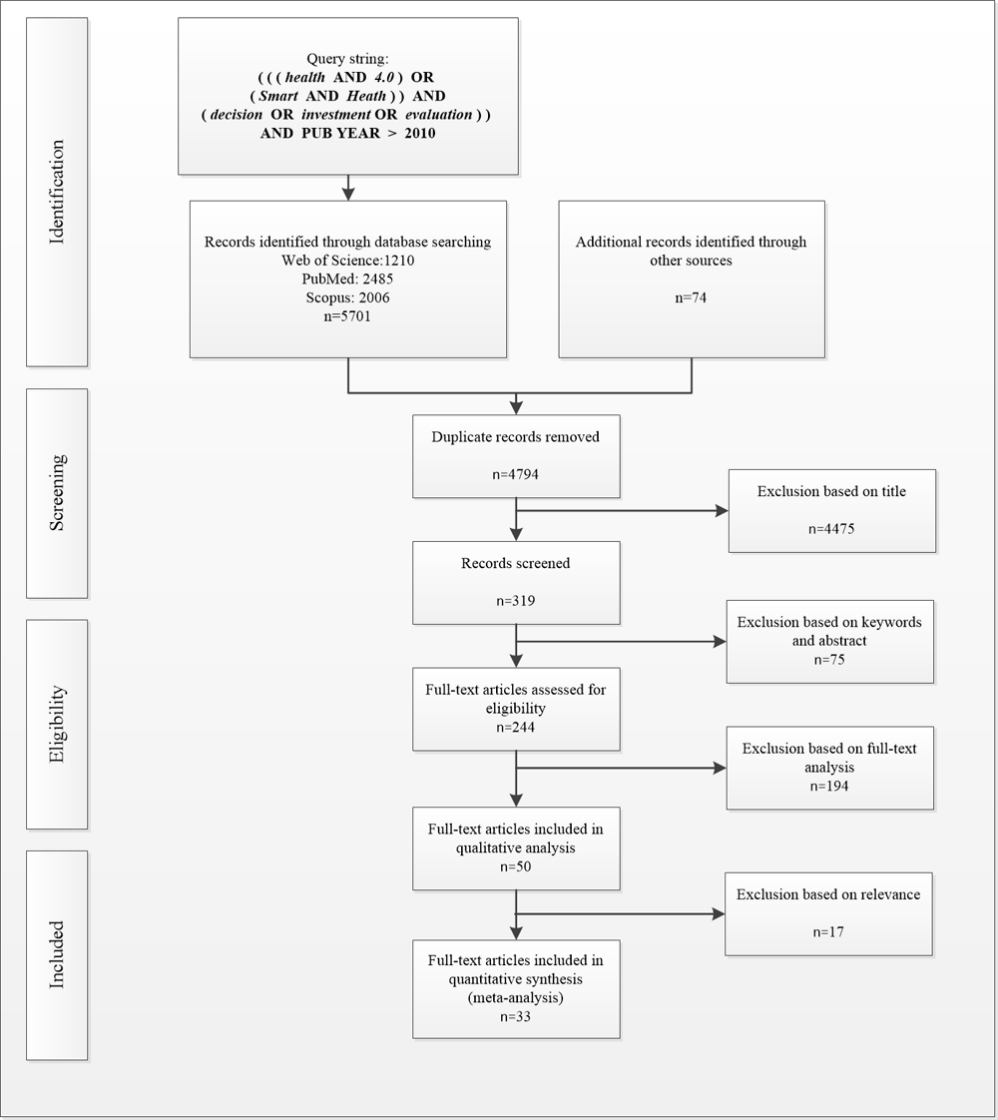
Selection of studies for the review.

In the second stage, we randomly selected five articles from each database to compare their keywords with those from the research dimensions used in the first stage [42]. The objective was to take into account the fact that different taxonomies may be associated with a given subject, potentially compromising the search. From the comparisons, we identified the need to add the keyword *smart* to our inquiry. A new search, with this keyword included, generated 74 additional papers, giving us a total of 5775 publications scattered among the databases, as shown in Figure 1. We conducted both search stages from July 2000 to August 2020. Figure 1 charts the process of the identification of relevant studies and the final selection of the studies included in the review.

### Selected Studies

The definition of different inclusion and exclusion criteria was post hoc because the researchers’ familiarity with the studies increased. In the first exclusion process (screening), we considered only articles in English published in peer-reviewed journals. We removed duplicate publications from the portfolio, reducing the number of articles from the initial 5775 to 4794. In the next exclusion step, the paper titles were individually verified to determine their alignment with the research topic. This resulted in 4475 papers being deemed irrelevant to the research. The remaining 319 articles that passed the title screening were then checked for the alignment of keywords and abstracts with the research topic. A total of 75 articles were excluded, resulting in 244 articles being considered in the eligibility step.

The next step was to determine the eligibility of the papers. Best practice guidelines for conducting scoping reviews recommend that 2 separate reviewers carry out the literature search and sifting process. They must both agree before the study can be included. Therefore, we took special care to ensure interrater reliability, with at least two separate reviewers involved in the process.

We carried out the two separate review processes and performed a full-text analysis of the 244 articles to determine their eligibility. In all, 50 articles were identified by both reviewers as fully aligned with our research interests. We then evaluated the papers keeping in mind the criteria of relevance and methodological rigor. In this process, we added a third reviewer, and a majority vote determined the inclusion of a paper. By the end of this stage, 30 articles were considered appropriate for inclusion in the review. We also analyzed the references of these articles to identify relevant studies not yet included in the portfolio, but none were found. However, based on experts’ recommendations (qualitative analysis), three articles were added to the portfolio, resulting in a final number of 33 studies in the publication portfolio, as displayed in Figure 1.

## Results

### Overview

We charted and interpreted critical data from the publication portfolio to establish the grounds for the subsequent analytical step [39]. We followed a descriptive-analytical method [17,43], providing a broader and meaningful view of all papers and collecting standard information from each study. Driven by our investigation’s research questions, we organized the articles in a spreadsheet that included the following information: authors, year of publication, journal, aims, type of technology, application focus (eg, hospital processes or health treatments), valuation methods, decision-makers, users, challenges, and opportunities.

Table 1 shows a basic descriptive numerical summary of the publication count per year. Three main characteristics are noteworthy. First, as expected, studies on the financial evaluations of H4.0 technologies are recent. Second, there has been a slight increase in the number of publications in recent years (2018-2020). Finally, the number of included articles is relatively small (n=33), which may be due to the novelty of H4.0 technologies and the multidisciplinary nature of the investment evaluation requirements and its complexity. These findings reinforce the convenience of using a scoping review approach.

**Table 1:** Number of publications per year.

Year	Number of publications
2011	2
2012	1
2013	2
2014	4
2015	4
2016	3
2017	3
2018	6
2019	5
2020	3

### Collate, Summarize, and Report Results

In this step, the results were collated, summarized, and reported based on a thematic framework such that a narrative account of the publication portfolio became available. Following the study by Levac et al [39], we carried out three complementary analyses to increase the consistency of this step. First, we performed a descriptive thematic analysis to collate and summarize the results. Second, based on the reported results, we developed a detailed analysis of the characteristics, contributions, and challenges of H4.0 technology evaluation tools. We report this analysis in the *Analysis of Results* section. In the *Classification Framework* section, we describe an emerging framework that synthesizes the empirical patterns of the analyzed papers. Finally, we discuss our findings’ implications in a broader context, ensuring the scoping study methodology’s legitimacy for both theory and practice [39]. In this discussion, we have also listed the research gaps and proposed research alternatives for future studies.

We now expand on the first step, providing detailed information on the characteristics of key publications. We conducted a word cloud analysis using the titles, keywords, and abstracts of papers in the portfolio. Multimedia Appendices 1-3 and Table 2 include the results, which provide initial evidence to answer the research questions. *Health* was the most frequent word, followed by *cost, cost-effectiveness*, *study, evaluation, care,* *patients,* and *data.*

The word cloud analysis anticipates the interdisciplinary nature of the manuscripts in the portfolio, allowing us to identify cost-effectiveness evaluation as the most recurrent. In addition, the incidence of the words *management, clinical,* and *patient* anticipates the need for health care institutions to incorporate a broad set of players in the investment decision process. We emphasize the absence of words such as quality, value, and bundle, which anticipate challenges and opportunities in current research on H4.0 technology investment analysis.

Table 3 reports the top 15 most frequent authors in the portfolio and the number of documents they authored, showing some of those who authored just one paper and the entire list of those who authored two or more. From the list of 146 authors, 1 participated in three studies, 2 participated in two studies, and the remaining 143 appeared in only one article. A large number of authors with small authoring prominence is typical of research topics about which knowledge is still incipient, such as H4.0 technology investment evaluation, reinforcing the convenience of adopting a scoping review as the methodological approach. We also observed a large average number of authors per publication (mean value of 4.33, SD 2.24), which is typical of publications in the medical field.

The journals’ analysis also reinforced the topic’s multidisciplinary nature. Table 4 reports the number of papers by category, and Table 5 reports the number of papers by journal. The Web of Science category *Health Care Sciences & Services* has the highest frequency of 16, followed by *Medical Informatics* (n=10), and *Pharmacology & Pharmacy* (n=2). The remaining categories displayed a frequency of 1 (6/9, 67% of the sample). Two journals published four manuscripts each: *Journal of Medical Internet Research* and *JMIR MHealth and UHealth*.

**Table 2:** Most frequent words in titles and abstracts.

Section and word			Count
**Title**			
	Health	12	
	Evaluation	10	
	Cost	8	
	Cost-effectiveness	7	
	Effectiveness	7	
	Study	7	
	Based	5	
	Decision	5	
	Economic	5	
	Management	5	
**Keyword**			
	Health	31	
	Results	26	
	Methods	24	
	Study	23	
	Analysis	20	
	Based	19	
	Care	19	
	Data	17	
	Background	16	
	Cost	16	

**Table 3:** Top 15 authors and frequency of appearance in publications (alphabetically ordered).

Author	Frequency
Wernz, Christian	3
Trajkovik, Vladimir	2
Zhang, Hui	2
Abrams, Keith R	1
A'Court, Christine	1
Adem, Abdu	1
Aloui, Saber	1
Augusto, Vincent	1
Babar, Zaheer Ud Din	1
Baio, Gianluca	1
Ball, Daniel R	1
Belani, Hrvoje	1
Berta, Whitney	1
Bertranou, Evelina	1
Beutner, Eric	1

**Table 4:** Frequency of manuscripts stratified according to Web of Science category.

Web of Science category	Frequency
Health care sciences and services	16
Medical informatics	10
Pharmacology and pharmacy	2
General and internal	1
Information systems	1
Computer sciences	1
Medicine	1
Multidisciplinary sciences	1
Operations research and management	1

**Table 5:** Frequency of manuscripts by journal.

Journal	Frequency
*Journal of Medical Internet Research*	5
*JMIR mHealth and uHealth*	4
*The Oncologist*	2
*Applied Health Economics and Health Policy*	1
*Biomedical Instrumentation and Technology*	1
*BMC Health Services Research*	1
*BMJ Open*	1
*Clinical Therapeutics*	1
*Frontiers in Pharmacology*	1
*Global Health Science and Practice*	1
*Health Affairs*	1
*Health Care Management Science*	1
*Health Economics Review*	1
*Implementation Science*	1
*Industrial Management and Data Systems*	1
*Information Systems and e-Business Management*	1
*International Journal of Medical Informatics*	1
*International Journal of Operations and Production Management*	1
*Journal of Multidisciplinary Healthcare*	1
*Journal of Operations Management*	1
*Journal of the Canadian Academy of Child and Adolescent Psychiatry*	1
*Journal of the Royal Statistical Society. Series A: Statistics in Society*	1
*PLOS ONE*	1
*Proceedings of the 51st Hawaii International Conference on System Sciences*	1
*Value in Health*	1

### Analysis of Results

Tables 6 and 7 summarizes the papers listed in the rows by year of publication and their different content characteristics. We started by reporting the type of technology analyzed, grouping them according to their role within the health care organization. The study by Aceto et al [5] proposed four interrelated subsets: (1) communication, (2) sensing, (3) processing, and (4) actuation. Communication involves different interactions and disseminating health-related information, supporting patient-professional relationships, and providing collaborative care. Related H4.0 technologies provide support to increase accessibility, exchange, and sharing of information. Sensing refers to acquiring information about a patient, equipment, material, or process without necessarily making physical contact with them. Processing refers to technologies that may change or process the acquired data, producing actual information in any manner detectable by an observer. Finally, actuation refers to technologies responsible for moving and controlling a system, mechanism (electronic or mechanical), or software based on the information and signals received.

**Table 6:** Classification of contents in the portfolio of papers.

Study and year	Non Healthcare 4.0	Healthcare 4.0			Technologies			Valuation methods						
		Sensing and communication	Processing and actuation	Stand alone		Bundle or portfolio	Deterministic			Uncertainty no option			Uncertainty option analysis	
							Cost		Value	Cost	Value	Cost		Value
Dreyfuss and Roberts [44] (2011)			✓^a^	✓										✓
Grutters et al [45] (2011)			✓	✓										✓
Marsh et al [46] (2012)	✓			✓										✓
Favato et al [47] (2013)				✓										✓
Drummond et al [48] (2013)	✓			✓			✓							
Pertile et al [49] (2013)	✓									✓				
Boydell et al [50] (2014)		✓		✓										
Kvedar et al [51] (2014)		✓	✓	✓		✓								
Wernz et al [14] (2014)		✓	✓	✓										✓
Atwood et al [52] (2015)		✓	✓	✓										✓
Wernz et al [13] (2015)		✓	✓	✓										✓
Gobbi and Hsuan [53] (2015)		✓		✓						✓				
Merlo et al [54] (2015)										✓				
Sharma et al [55] (2016)		✓	✓			✓								
Matthew-Maich et al [56] (2016)		✓	✓	✓										
de Grood et al [37] (2016)		✓		✓										
Lavallee et al [57] (2017)		✓				✓								
Kim and Lee [58] (2017)		✓	✓	✓										
Rejeb et al [59] (2017)	✓					✓				✓				
Greenhalgh et al [60] (2017)		✓	✓	✓			✓							
Long et al [61] (2018)		✓				✓								
Adjekum et al [62] (2018)		✓		✓										
Winters et al [63] (2018)		✓		✓										
Baines et al [64] (2018)		✓	✓	✓										
Taj et al [65] (2019)		✓	✓	✓										
Dogba et al [66] (2019)		✓		✓										
Loncar-Turukalo et al [67] (2019)		✓	✓	✓		✓								
Shahid et al [68] (2019)		✓	✓	✓										
Wüller et al [69] (2019)		✓	✓	✓										
Chouvarda et al [70] (2019)		✓		✓						✓				
Hasselgren et al [71] (2020)		✓												
Peng et al [72] (2020)		✓	✓	✓										
Ismail et al [73] (2020)		✓	✓											

^a^Present in study.

**Table 7:** Classification of some contents in the portfolio of papers.

Study and year	Decision maker				User		
	Medical	Administrative	Patient	Medical		Administrative	Patient
Dreyfuss and Roberts [44] (2011)		✓^a^				✓	
Grutters et al [45] (2011)		✓				✓	
Marsh et al [46] (2012)		✓				✓	
Favato et al [47] (2013)	✓	✓					
Drummond et al [48] (2013)	✓			✓			
Pertile et al [49] (2013)		✓				✓	
Boydell et al [50] (2014)	✓		✓				✓
Kvedar et al [51] (2014)	✓		✓				✓
Wernz et al [14] (2014)	✓	✓				✓	
Atwood et al [52] (2015)	✓	✓				✓	
Wernz et al [13] (2015)		✓				✓	
Gobbi and Hsuan [53] (2015)		✓				✓	
Merlo et al [54] (2015)	✓	✓	✓	✓		✓	
Sharma et al [55] (2016)	✓			✓			
Matthew-Maich et al [56] (2016)	✓	✓		✓			
de Grood et al [37] (2016)	✓	✓		✓		✓	
Lavallee et al [57] (2017)	✓			✓			
Kim and Lee [58] (2017)	✓		✓				✓
Rejeb et al [59] (2017)	✓			✓			
Greenhalgh et al [60] (2017)			✓				✓
Long et al [61] (2018)	✓			✓			
Adjekum et al [62] (2018)	✓			✓			
Winters et al [63] (2018)	✓		✓	✓			✓
Baines et al [64] (2018)	✓		✓	✓			✓
Taj et al [65] (2019)	✓			✓			
Dogba et al [66] (2019)	✓		✓	✓			✓
Loncar-Turukalo et al [67] (2019)	✓			✓			
Shahid et al [68] (2019)	✓			✓			
Wüller et al [69] (2019)	✓			✓			
Chouvarda et al [70] (2019)	✓			✓			
Hasselgren et al [71] (2020)	✓	✓		✓		✓	
Peng et al [72] (2020)	✓			✓			✓
Ismail et al [73] (2020)	✓	✓		✓		✓	

^a^Present in study.

There may be overlaps among the technology subsets. Following the classification in the study by Tortorella et al [1], we further grouped H4.0 technologies into two bundles according to their role within the hospital: sensing-communication (reported under the column labeled *sensing* *and communication*) and processing-actuation (reported under the column labeled *processing and* *actuation*; Table 6). Consistent with previous studies’ reports on the incidence of technological applications (eg, the study by Tortorella et al [1]), the number of articles evaluating sensing-communication is significantly greater than those analyzing processing-actuation. In addition, and somewhat paradoxically, given the nature of H4.0 technologies, most studies focus on just one technology, with only six manuscripts addressing bundles of technologies.

Regarding the thematic analysis (data not included in Table 6 because of space limitations), we observed two groups of studies on H4.0 technology evaluation in health care organizations: those related to health treatments and those related to hospitals’ supporting and administrative processes. Articles in the former group were relatively more frequent than those in the latter group.

The evaluation of different technologies contributes to health improvement in various ways. H4.0 technologies contribute to reductions in diseases such as cancer [44,59] and allow for better connectedness that manages individual and community health holistically by leveraging various technologies [70]. Connectedness can also incorporate telehealth and integrated care services, covering the entire spectrum of health-related services that address healthy individuals and patients with chronic conditions [70]. In addition, neural networks improve decision-making, improving care delivery at a reduced cost [68].

The aforementioned analysis allowed us to describe the types of technology and the health improvement sought from their use. Next, we addressed the first research question. For this purpose, we surveyed the methodologies that health care institutions reportedly use for evaluating investments in H4.0 technologies.

With regard to the different methodologies for evaluating investments in H4.0 technologies, of the 33 papers analyzed, only 14 (42%) presented valuation methods, whereas 7 (21%) focused on cost valuation methods and 7 (21%) focused on value methods. Regarding forms of considering uncertainty in the analysis, of the 33 papers analyzed, 2 (6%) used deterministic techniques that disregarded uncertainties, 5 (15%) accounted for uncertainty but did not use real options, and 7 (21%) accounted for uncertainty using a real-options approach.

As we can observe, studies that consider the cost implications of investing in H4.0 technologies focus on economic analysis, adopting a cost-effectiveness and cost-minimization perspective. These studies were complemented by the application of a Bayesian sequential economic evaluation model for health technologies in which an investigator has flexibility over the timing of a decision to stop carrying out research and conclude that one technology is preferred over another on cost-effectiveness grounds [49]. A total of five manuscripts took a real-options perspective that incorporates value considerations but refers to past work, mainly published at the beginning of the time window of analysis.

The portfolio of 33 studies lists three types of decision-makers, who may be consulted individually or in groups: doctors, administrative staff, and patients. Doctors appear in 82% (27/33) of the studies; 39% (13/33) incorporate the administrative perspective; and 21% (7/33) contain the patient perspective. Although there is a dominance of expert opinion based on medical advice, the variety of decision-makers is a positive result that further claims support for a multidisciplinary analysis that incorporates the different types of users affected when evaluating investments in H4.0 technologies. Users of the information derived from the evaluations are also doctors, administrative staff, and patients; however, administrative users are predominant because they are direct users of the economic information.

A relevant aspect of the 33 studies analyzed in the portfolio is that 48% (16/33) of them present results of scoping or systematic literature reviews and meta-analyses (1 meta-analysis present among the 16 papers). However, they focused on the medical convenience of H4.0 technology investments, not on exploring specific economic evaluation tools, and mainly assessed a particular technology (eg, physicians’ adoption of eHealth technology or smart device apps for older adults).

We were able to consolidate several relevant propositions for the economic evaluation of H4.0 technologies. A fundamental contribution of our review is the identification of the main antecedents of hospital investment decisions in technology, such as the health care system, the socioeconomic and cultural context, and its mission [13,14]. Regarding the health care system, the findings emphasize the role of health insurance coverage, financing methods, reimbursement methods for hospitals, methods used to make payments to physicians, and hospital ownership as antecedents of H4.0 technology investments. The existence of these antecedents anticipates the challenges of investment evaluations [14,37,44-74].

The appropriate deployment of medical technology should help to contribute to the quality of health care delivered, improve access to information, and contain costs [52]. Among the most promising evaluation alternatives is the framework in the study by Greenhalgh et al [60] to assist implementation teams in identifying, understanding, and addressing the interacting challenges to achieving sustained adoption, local scale-up, distant spread, and long-term sustainability of their technology investments in hospitals. Complementing this analysis is the call for applying a simple, multiattribute rate technique in the valuation process, as proposed in the study by Wernz and Zhang [13].

We identified four main challenges faced by health care institutions when evaluating investments in H4.0 technologies. First, H4.0 technologies should be analyzed as a bundle of technologies rather than individual solutions. As proposed in the study by Aceto et al [5], there are four overlapping groups of technologies based on their roles and applicability within the hospital. In our portfolio, of the 33 papers, only 6 (18.1%) analyzed H4.0 technologies as a bundle. Second, as mentioned earlier, there is a research gap in valuation methodologies for H4.0 technologies, especially in the realm of real-options analysis. Third, regarding who makes the decision to acquire the technology (medical personnel, administrative staff, or patient), 82% (27/33) of studies focused on the medical personnel as the main decision-makers. In contrast, only 24% (8/33) focused on patients, and a single paper integrated the 3 actors in the process [54]. Fourth, regarding the user of the technologies, 67% (22/33) of studies focused on medical personnel, whereas 24% (8/33) indicated that the main user was the patient.

Real-options strategies offer a transparent method for weighing the costs and benefits of adopting and further researching new and expensive technologies [44,45]. Such valuation methodologies incorporate the value of future new information in the current analyses. The articles in the portfolio report real-options applications in proton therapy adoption analysis [44,45] and help formulate better human papillomavirus vaccination strategies [47]. Surprisingly, none of the articles using real-options analysis incorporated uncertainty correlations among the bundles of technologies. This is a critical shortcoming given the antecedents that report the importance of taking broader portfolio considerations when evaluating related and uncertain investments in areas such as biotechnology research and development [75].

Health care managers often make purchasing decisions without adequately assessing the resource demands, up-front costs (including integration costs), workflow impact, reimbursement potential, and other factors needed to fully understand the value added by new medical technology [52]. Consequently, health care authorities may risk failing to conduct thorough due diligence before purchasing medical technology. Under these circumstances, organizations might add unnecessary costs to their budget without adding significant clinical or operational value.

Selecting new medical technology for a health care organization can be a daunting task. It is crucial to implement a systematic approach for evaluating the latest medical technology, starting with a clearly articulated need for the technology. If organization authorities are unwilling to assess and redesign processes to fully use the new medical technology, investment withholding may be the most suitable course of action. Moreover, there is a risk of bias in purchasing the latest technology simply because it is available [52]. Overall, health care organizations rarely assess a systematic decision process that considers all organizational objectives and analyzes and integrates comprehensive data [52].

Providing universal access to innovative, high-cost technologies has led to tensions in today’s health care systems. The stress becomes particularly evident in the context of scarce resources, where the risk of taking contentious coverage decisions increases rapidly. If health care institutions intend to maintain sustainable access to H4.0 technologies in the future, new approaches are needed to reconcile these different perspectives [48]. Overall, although policy makers request rapid and at-scale technology implementation, the reality is that when dealing with the multiple complexities of health and care, it is challenging to go beyond small-scale demonstration projects [52]. To address the need for new approaches, we propose in the next section a framework for the evaluation of H4.0 technologies in hospitals.

### Classification Framework

Scientific research presents frameworks because managers use them to support their analysis and provide validity to the decision-making process [76]. We developed an emerging framework from the study we conducted on the research on hospital evaluations of H4.0 technologies.

Frameworks have multiple advantages. They decrease the number of uncertainties when addressing a new phenomenon, as is the case with H4.0 technologies. Frameworks can support the selection of investment strategies. In addition, frameworks can depict features of various phenomena [77], compare and guide numerous organizational practices [78], support the execution of tasks [79], and refute or confirm a particular management approach [80].

When developing the framework, it is fundamental to determine the rationale that validates the theoretical process. Given the scoping review’s multidisciplinary and integrative nature, we have chosen a process of abstraction, that is, we obtain higher-order themes from lower-order elements [81]. Therefore, we follow the most common abstraction process, in which lower-order themes are a function of the findings of individual studies, and higher-order structures link and organize the lower-order themes [81]. Such a method should result in the advancement of knowledge rather than a simple overview or description of a research area [82], that is, it should not be descriptive or historical but should preferably generate a new conceptual framework. In addition, we checked the reliability of higher-order themes using a focus group of experts. It is worth noting that the higher-order themes respond to taxonomy and not from a typological process [82,83].

**Figure 2:**
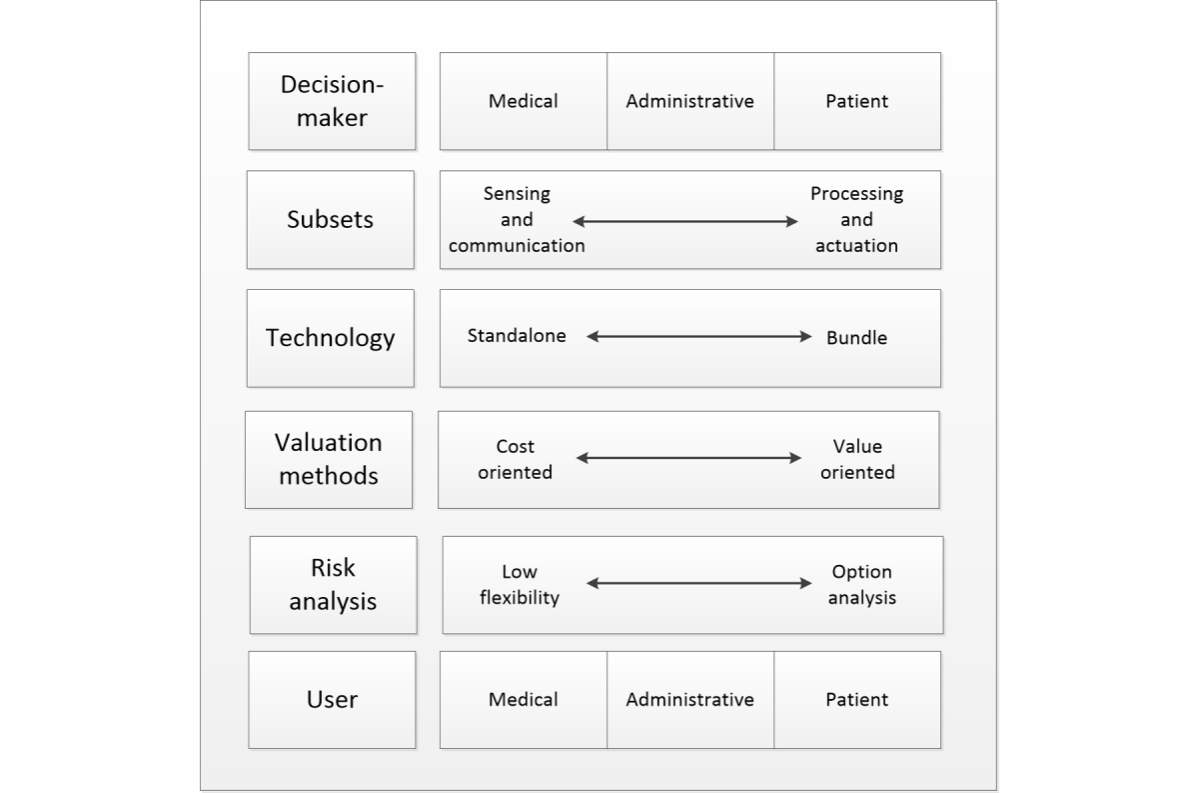
Classification framework.

Figure 2 presents the proposed classification framework. It focuses on the most fundamental tensions that organizations face when analyzing H4.0 technology investments and reflects the most prominent features of our publication portfolio. We categorized the type of technology analyzed based on its focus, sensing-communication or processing-actuation, following the classification in the study by Tortorella et al [1]. In this process, we classify lower-order themes into higher-order classification. We describe the number of technologies evaluated, depending on whether the analysis refers to stand-alone technologies or bundles. We also report the evaluation method, stating whether it is based only on cost or also takes into account value considerations. We considered whether the analysis does not incorporate flexibility in the valuation process or explicitly incorporates it using a real-options approach. We also examine the portfolio of manuscripts regarding the variety of decision-makers included and the type of technology users. For all these cases, we propose higher-order themes for the portfolio of manuscripts.

The framework not only helps to classify a particular research paper but also has utility for practice. It may allow hospital authorities to understand what type of organizational process they have in place to analyze investment decisions in H4.0 technologies. In addition, it helps to anticipate the complexity of the task. When reflecting on the most critical tensions that hospitals face, the structure would allow authorities to detect the underlying leadership and change-management challenges.

When categorizing the portfolio of manuscripts using the proposed framework, we identified a significant concentration of studies on the left side. It seems reasonable to observe such an unbalanced distribution, given the developing nature of H4.0 technologies. However, it also signals an essential shortcoming of the current studies, directing further research propositions. There is a risk that hospitals might have been making decisions by following isomorphic behavior [84], which is not necessarily the best rational approach. Research concentration might reflect herding behavior in which hospitals imitate one another instead of following a robust, innovative path.

**Figure 3:**
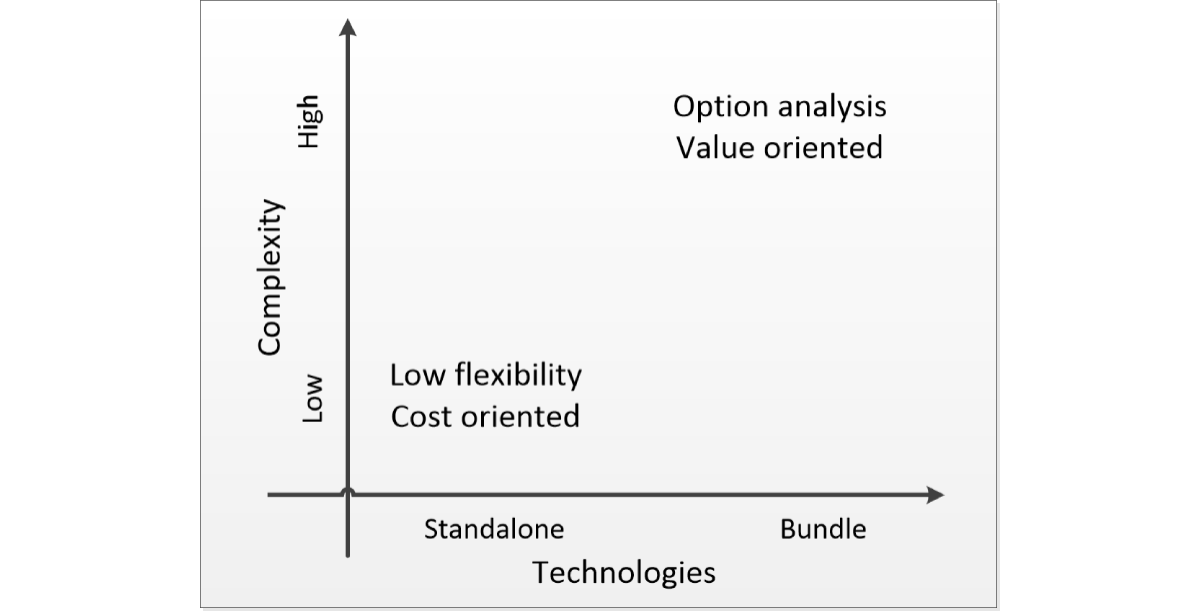
Research opportunity map.

We further analyzed the framework and developed a research opportunity map, displayed in Figure 3, focusing on two dimensions of the framework: the complexity of the analysis and the number of technologies considered.

From the research map, it is possible to indicate that there is a research opportunity related to analyzing bundles of technologies with complex relationships that incorporates uncertainty correlations. It is important to emphasize that complex relationships do not necessarily imply more complex analyses. The challenge is to integrate a higher level of complexity with straightforward analytical tools. We will return to this point at the end of the following section.

## Discussion

### Principal Findings

This section addresses the third research question, that is, we identify the crucial characteristics that methodologies for evaluating investments in H4.0 technologies should have. These characteristics also represent research gaps that should be addressed in future research.

Overall, we anticipate that the evaluation of H4.0 technologies presents challenges and opportunities that are similar to those related to general technology investments, although the evaluation is more complex because of the nature of H4.0 technologies. We also observe that the existing research does not entirely succeed in helping hospitals in the investment decision-making process, leading to promising research opportunities.

### Insufficient Economic Valuation of H4.0 Technology Investments

Decades of research on health technology assessment have resulted in a framework that includes economic evaluation as a fundamental pillar. However, studies that rigorously integrate this economic perspective are still scarce. Advancements focus more on cost-effectiveness than economic value at the public policy level rather than at the hospital level. In addition, studies have confirmed that health care institutions rarely apply a systematic analysis that considers all organizational objectives and integrates comprehensive data [13]. For instance, the relatively low commercial externality valuation is one of the shortcomings of economic analysis.

Although some older studies on real options include cost and value considerations, more recent propositions tend to overfocus on cost analysis, imposing a challenging bias on investment decisions. Among propositions that incorporate value, the net present value analysis is the most frequently used, often resulting in a suboptimal decision because it does not consider the value of future options and managerial flexibility [13]. Usually, simple cost-benefit analysis and subjective assessment replace sophisticated analytical methods and objective data at the risk of not investing in more expensive technologies with higher health impacts because of their investment requirements. The development of real-options approaches that include value considerations targeted at evaluating investments in H4.0 technologies is a promising research opportunity that should resonate positively among practitioners.

### Explicit Assessment of Technological Interrelationships

The literature provides evidence that for maximizing the return on H4.0 technology investments, hospitals should consider them in bundles. Studies have proposed distinct bundles (or groupings) of H4.0 technologies. Sharma et al [55] categorized technologies into three bundles according to the extent of patient-centered integration and caregiver interaction. The study by Aceto et al [5] conceptually proposed four overlapping groups of technologies based on their roles and applicability within the hospital. The study by Gastaldi and Corso [85] proposed another categorization of H4.0 technologies, dividing them into four macroareas, further subdivided into 14 solutions provided by each technology. Finally, the study by Alrige and Chatterjee [86] suggested a taxonomy to classify wearable technologies in health care systems according to three major dimensions: application, form, and functionality.

Although the literature still lacks consensus on the correct taxonomy of bundles of H4.0 technologies and how to combine them to act synergistically, it is clear that the valuation should incorporate the bundling of technologies. Therefore, researchers and institutions need to assess portfolio effects explicitly [75]. The literature on real options includes several studies that explicitly address portfolio considerations [75,87-89], providing a potential area of extension to H4.0 technology investments. In analyzing hospital investments, research incorporating portfolio considerations is scarce (eg, the study by Wernz and Zhang [13]) and does not include real-options valuations. It is fundamental to understand whether investing in technology bundles creates super- and subadditivity [75], altering the net economic contribution of different alternatives and eventually changing the suggested priorities.

We detected recent efforts to provide an accessible and usable framework that would enable multiple objectives, mainly developed by authors seeking to design, develop, implement, scale-up, spread, and sustain technology-supported health or social care programs to identify and help address the critical challenges in different domains and the interactions among them [51,60,67]. However, the developments only start to address the shortcomings identified in our scoping review, opening opportunities for future research.

### Incorporate Fundamental Uncertainties

H4.0 technologies enhance efficiency and quality in health care systems. However, fundamental uncertainties exist in the definition of industry standards for many of these technologies, creating uncertainty when evaluating investments. Factors that add additional complexities to technological advancements relate to uncertainty regarding patient demands and competition [13].

To reduce the risk of investing in a technology that ends up being crowded out and not adopted as the standard, hospitals have several alternatives; further research is needed to explore their viability. Surprisingly, the discussion about standards is scarce in the economic evaluation of H4.0 technologies, with the main focus continuing to be on their efficacy.

### Integrating Administrative, Medical, and Patient Perspectives in the Evaluation Process

The fourth research opportunity relates to integrating medical, patient, and administrative considerations in the valuation process. We have already stressed that the interrelationships among technology bundles incorporate nontrivial challenges. In addition, institutions should consider the risk of investing in technologies that fail to establish the industry standard. The final layer should adequately balance medical benefits with economic costs. It is still unclear how to achieve such reconciliation [48]. The central problem concerns the resolution of the economic logic versus medical logic debate. On the one hand, doctors favor technologies with the most promising medical effects, regardless of uncertainty and varying requirements of investment and cost. On the other hand, the administrative staff need to ensure the hospital’s economic viability. Amid high levels of uncertainty, the amount of investment and the operating costs, that is, the economic logic, might contradict the medical logic. Research is needed to explore the most suitable ways in which hospitals can coordinate both perspectives.

In integrating the different perspectives into the valuation process, hospitals need to include those of the patient for at least three reasons [48]. First, a comprehensive assessment should consider patients’ views on the satisfaction and acceptability of health technologies. Second, with chronic forms of disease and disability, patients and their families play a more active role in health care decisions. Patients’ lifestyles and behaviors may dramatically influence long-term prognoses of chronic conditions. Third, the involvement of patients increases transparency and openness in public policy [48]. We acknowledge that incorporating the patient’s view in the investment decision analysis adds a layer of complexity to a process that is already difficult to manage. However, any valuation analysis that considers costs and value without including the patient perspective will be incomplete.

The integration of different perspectives provides an opportunity to cross-fertilize research on H4.0 technology investments with adaptive leadership tools [90]. Alternatively, the incorporation of H4.0 technologies equals establishing a dynamic organizational capability that demands from employees the ability to leverage interpersonal relationships conducive to productive dialog [91].

### Remain Manageable in the Decision-Making Process

Previous studies describe hospitals’ investment decisions as ad hoc, informal, political, without sufficient data analysis, and not aligned with the institutions’ mission and strategy [13]. We argue in favor of assessments that explicitly consider technological interrelationships, incorporate fundamental uncertainties, and integrate administrative and medical insights. However, our argument comes with an essential caveat: analytical methods should avoid introducing complex evaluation tools that hamper the hospital’s decision-making process.

At first, such a requirement seems to be challenging. We have suggested incorporating bundles of technologies, mapping multiple uncertainties, considering value implications and not exclusively cost aspects, and including different stakeholders’ perspectives. A priori, these requirements go against the simplification of the decision-making process. However, it might be possible to solve this tension by articulating the valuation process in different stages. We envision a lean financial valuation that combines these competing demands without drastically complicating the decision process.

The lean financial valuation of H4.0 technology investments involves simplifying, streamlining, and harmonizing essential valuation processes to create a leaner, more efficient valuation operation. The current research opportunity relates to developing lean organizations that incorporate valuation tools that simultaneously address challenges such as complex uncertainty relationships and bundle effects into organizational structures that adjust to lean principles.

### Limitations and Final Remarks

This study examined how hospitals approach investment decisions in H4.0 technologies by using a scoping review of the existing literature. We performed a search for journal articles in four databases and screened relevant contributions to consolidate a publication portfolio on the topic, following predefined criteria. The results of the scoping review were explored using the following stepwise approach:

1. A descriptive numerical summary and thematic analysis.
2. Identification of trends and challenges in H4.0 technology investment evaluation.
3. Proposal of a classification framework for H4.0 technology investment evaluation.
4. Identification of research opportunities and proposals for future research directions from a hospital investment management point of view.

Despite the topic’s recency, we observed that research in H4.0 technologies expands interdisciplinarily with a diversified set of applications and functionalities. In terms of the economic evaluation, studies on H4.0 technologies tend to overfocus on cost considerations and underemphasize cost-value relationships. Studies that consider both sides of the economic valuation (ie, value and cost) use real-options analysis and tend to be older in the sample of studies analyzed. Although the impacts of H4.0 technology adoption substantially increase when hospitals adopt technologies in bundles, research mainly focuses on the analysis of single technologies. Finally, recent studies have called for the integration of different actors in the decision process by developing a comprehensive, consistent, and data-driven framework for evaluating hospitals’ investment decisions. We have proposed a framework that serves as a starting point.

Our study includes some noteworthy limitations, mostly related to its nature and methodological choices. As Industry 4.0 was formally acknowledged in 2011 and H4.0 is a concept derived from it, our scoping study only encompassed studies after that year. However, it is worth mentioning the existence of initiatives aimed at valuing Industry 4.0 technologies in health care systems not characterized as such and dating earlier than 2011, which is a limitation of our research. Nevertheless, because studies before 2011 were scarce and scattered, and the number of publications on the topic has significantly increased in the past few years, we believe that our choice of the search period returned all relevant studies on H4.0 technologies.

A second limitation is that we focused our literature analysis and discussion on H4.0 technology evaluation within hospitals. However, the concept of health care has expanded beyond the limits of health care organizations (ie, hospitals and clinics). In fact, with the advent of *smart cities*, complementary aspects of health care have been integrated because of the increased level of interconnectivity and data acquisition, allowing health care services to be demanded remotely. Our study did not analyze these aspects and exclusively considered hospitals the units of analysis.

Third, it is worth emphasizing that we combined insights from two perspectives to develop the proposed framework: the state of the practice at hospitals and the state of the art in the literature. However, our main focus was on research, and we did not include a specific survey of empirical studies mapping hospital tools. This is simultaneously a limitation of our investigation and a research opportunity.

Finally, identifying trends, challenges, and theoretical gaps through this scoping review allowed us to develop a framework. However, we acknowledge that this is the first step toward the proposal of an in-depth framework. Future studies could use the theoretical consolidation of the studies in our paper as a conceptual baseline for developing such a detailed H4.0 technology evaluation framework. We hope that our classification framework will act as a solid starting point for future developments in evaluating H4.0 technology investments.

## Appendix

Multimedia Appendix 1 Word cloud analysis using titles as input.

Multimedia Appendix 2 Word cloud analysis using keywords as input.

Multimedia Appendix 3 Word cloud analysis using abstracts as input.

## Abbreviations

H4.0: Healthcare 4.0
