# Supplementary figures and images for: Hospital Investment Decisions in Healthcare 4.0 Technologies: Scoping Review and Framework for Exploring Challenges, Trends, and Research Directions
Source: J Med Internet Res. 2021 Aug 26;23(8):e27571. doi: 10.2196/27571 (PMC8430851; doi:10.2196/27571)

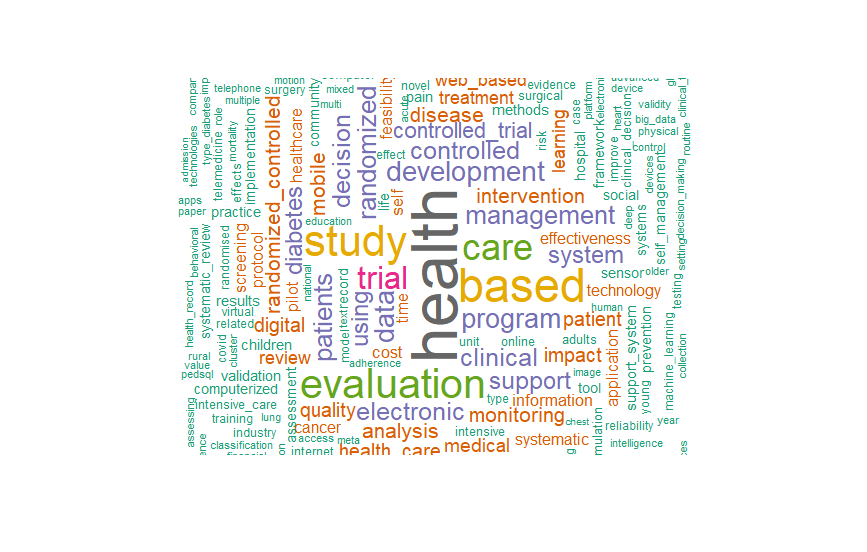

Supplement: Multimedia Appendix 1 [file jmir_v23i8e27571_app1.png]

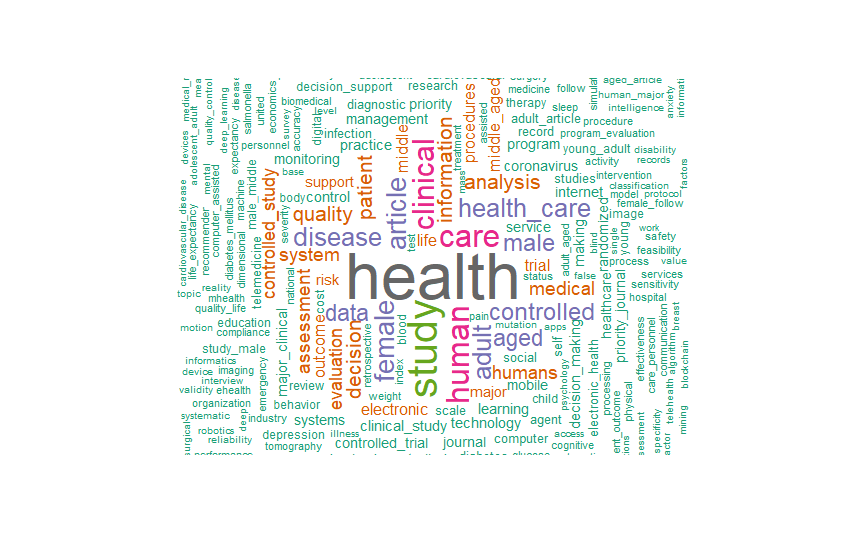

Supplement: Multimedia Appendix 2 [file jmir_v23i8e27571_app2.png]

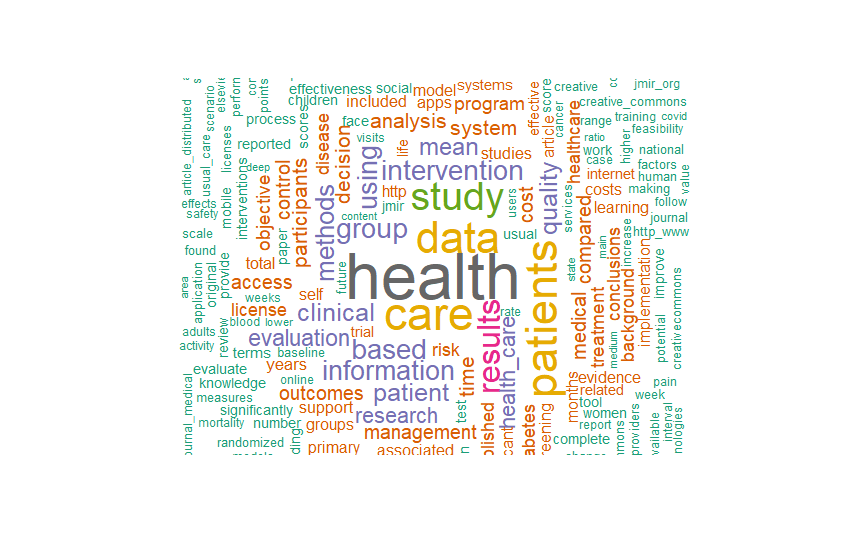

Supplement: Multimedia Appendix 3 [file jmir_v23i8e27571_app3.png]
